# Characterization of adolescent sexual practices*

**DOI:** 10.1590/1518-8345.6289.3711

**Published:** 2022-09-22

**Authors:** Nathalia Santarato, Nayara Gonçalves Barbosa, Anderson Lima Cordeiro da Silva, Juliana Cristina dos Santos Monteiro, Flávia Azevedo Gomes-Sponholz

**Affiliations:** 1 Universidade de São Paulo, Escola de Enfermagem de Ribeirão Preto, Centro Colaborador da OPAS/OMS para o Desenvolvimento da Pesquisa em Enfermagem, Ribeirão Preto, SP, Brasil.; 3 Universidade Federal de Juiz de Fora, Departamento de Enfermagem Materno-Infantil e Saúde Pública, Minas Gerais, Brasil.; 4 Universidade de São Paulo, Escola de Enfermagem de Ribeirão Preto, Centro Colaborador da OPAS/OMS para o Desenvolvimento da Pesquisa em Enfermagem, Departamento de Enfermagem Materno-Infantil e Saúde Pública, Ribeirão Preto, SP, Brasil.

**Keywords:** Adolescent Health, Reproductive Health, Health Education, Sexually Transmitted Diseases, Use Substance, Primary Care Nursing

## Abstract

**Objective:**

to characterize adolescents’ sexual practices and their association with sociodemographic variables, sources of information and behavioral habits.

**Method:**

a descriptive, observational, cross-sectional study conducted with 85 adolescents from public elementary and high schools in a city in the state of São Paulo. Data were collected through a structured, self-administered and anonymous questionnaire. Statistical analysis was performed using the χ2 test and Fisher’s test.

**Results:**

21.2% had started their sexual life through oral sex, with a predominance of females (94.4%), self-reported brown color (55.0%). The practice of vaginal sex was reported in 31.8%, with a mean age of initiation at 14.5 years. The female sex was predominant (77.0%), with a self-reported brown color (40.0%). The practice of anal sex was detected in 7.1%, with a mean age of 14.4 years, prevalent in females (83.3%), with a self-reported black color (50.0%). There was an association of alcohol, drugs and tobacco use with sexual practices (p<0.05).

**Conclusion:**

a diversity of sexual practices associated with substance use was detected, emphasizing the importance of the nurse’s role in planning and carrying out health education interventions with adolescents and families.

## Highlights

(1) Analysis of the sequence and time of initiation of sexual practices, demonstrating the diversity of sexual activities performed by adolescents.

(2) Family context and low openness to dialogue and its correlation with the initiation of adolescent sexual practices.

(3) Behavioral habits: association of alcohol, tobacco and drug consumption by adolescents with sexual activities.

## Introduction

Adolescence is an intermediate stage of human development that comprises the second decade of life. In Brazil, the Ministry of Health considers for adolescence the same age group prescribed by the World Health Organization (WHO), corresponding to the period from 10 to 19 years old^1^.

This life stage is surrounded by doubts and concerns, especially regarding current choices, perspectives for the future and self-care. Adolescence is a period marked by increased autonomy, social immaturity and risky behaviors that can have repercussions on the adolescents’ sexual and reproductive health, with a greater risk of unplanned and unprotected sexual practices, acquisition of sexually transmitted infections (STIs), unwanted pregnancies and unsafe abortions^2^.

Caring for adolescent health in an integral approach is a challenge, as this is a group in constant change and in the process of acquiring awareness of themselves and their insertion in society^1^. Adolescents are expected to have universal access to sexual and reproductive health services and actions, including reproductive planning and quality information on sexual education^3^. Sex education is essential for adolescents to acquire reliable information about sexuality, sexual health and safe practices. Armed with this information and recognizing the presence or absence of a support network formed by reference adults (family members, teachers and/or health professionals), they are able to safely initiate sexual practices and experience sexuality in a healthy way.

There are situations that affect adolescents’ sexual health and interfere unfavorably with the safe initiation of sexual life, such as precarious lifestyle, gender inequities, silencing, denial of sexual rights, disqualified information, and social and economic inequalities. These demand a careful consideration from adults and a multidisciplinary approach in terms of offering effective and lasting health actions that make sense for adolescents and allow them to develop autonomy over their care^4^.

The early initiation of sexual life (before age 15) is related to risky sexual behaviors, exposing adolescents to greater chances of acquiring STIs, having more sexual partners, making inconsistent use of contraceptive methods, getting pregnant without wanting to, and having sex with risky partners. Unprotected sexual practices entail risks for adolescents, such as the acquisition of STIs^5^ ^)^ and teenage pregnancy, which represent a multidimensional public health problem. It is thus necessary to understand the multicausality involved in adolescents’ sexual health in order to propose interventions in tune with reality and sensitive to their specificities^6^.

According to the National School Health Survey (PeNSE), about 28.0% of Brazilian schoolchildren in the 9th year of elementary school (average age of 14 years) have already had sexual intercourse^7^. Among sexually active adolescents, 24.7-30.8% reported that they did not use condoms in their last sexual intercourse^5^ ^),(^ ^7^. In Brazil, the percentage of live births to adolescent mothers is approximately 17.5%, with a heterogeneous spatial distribution in the territory, especially in regions with lower human development indices, which highlights the country’s severe regional inequities and the perpetuation of intergenerational cycles of poverty^8^.

In view of this scenario, the following question was asked: what factors influence adolescents’ sexual practices? In this direction, the present study aimed to characterize adolescents’ sexual practices and their association with sociodemographic variables, sources of information and behavioral habits. The results of this study may help to understand adolescents’ sexual practices and to guide intervention strategies in health education at school and in the community.

## Method

### Study type

This is an epidemiological, observational, cross-sectional study with a quantitative approach, conducted with 85 adolescents sequentially recruited from two state public schools in the city of Ribeirão Preto-SP, in 2019 and 2020.

This study is a part of a larger research project entitled “Sexual practices of adolescents in public schools in Ribeirão Preto-SP: prevalence, risk factors and social determinants” and was developed as a scientific initiation funded by the FAPESP agency Process No. 2019/11185-8.

### Data collection location

Data collection was carried out in two state public schools in the city of Ribeirão Preto-SP. School 1 was chosen among the other state schools in the municipality, as it was an empirical field for carrying out other research and extension activities of the research group in which the authors participate. Due to this previous contact, we had participative feedback from students and teachers. In order to choose school 2, we looked for similarity in population, number of students and social and economic level with school 1. The coordination of school 1 participated in the identification and articulation with school 2. Afterwards, the research project was presented to the board of school 2 and authorization was granted to continue the research data collection.

### Population and sample

The reference population consisted of all adolescents who were attending the elementary and high school grades of the two schools, regardless of grade, as long as their age coincides with that defined by the WHO and the Ministry of Health^1^. Participants were selected according to a convenience sample and based on the inclusion criteria: adolescents enrolled in elementary school II or high school and aged between 10 and 19 years.

### Data collection

Following the form of recruitment used in a previous similar study^9^, an agreement was first made between the researchers and the school directors. From then on, the researchers made a series of visits to the schools at the end of classes, with the students still in the classroom, so that the project could be presented to the adolescents. Potential participants, identified through the established criteria, were invited to participate in the research, informed that they should take the Free and Informed Consent Form (ICF) to their parents or guardians so that they would authorize their participation in the research. After being aware of the research and ethical aspects, the adolescents who returned with the signed informed consent and agreed to participate signed the Free and Informed Assent Term (TALE). If the possible participant was identified as over 18 years of age, the ICF was presented to them.

Only one person conducted the data collection, which took place in the schools themselves, in rooms designated by the board during school hours, and lasted an average of 20 minutes. This ensured that all data were collected in the same way, following the same understanding of what was being collected. The researcher remained in the same room as the adolescent during data collection, to ensure that participants had no doubts about what was being asked.

For data collection, an instrument developed and based on guidelines about good practices in conducting observational studies was used^10^, based on the authors’ professional experiences, and based on national and international scientific publications on the subject about adolescents’ risk factors^9^, sexual practices^11^ ^)^ and habits^5^ ^),(^ ^7^ ^),(^ ^11^ and thus provide a basis for achieving the objectives. It is a self-answered, semi-structured and anonymous questionnaire, in the form of a checklist, containing 33 questions divided into five structured blocks. In the present focus of the larger project, the fifth block, referring to contraceptive methods, was not used. The first block contains 7 questions that address sociodemographic data of participants and family members and housing conditions. The second block contains 6 items on adolescents’ habits and behaviors and family interactions. The third block contains 12 items that address information about sex education (family and school), and the fourth block contains 8 questions about adolescent sexuality.

### Study variables

The variables analyzed were: sociodemographic variables (age in years, sex, self-reported color, current job, location, type and material of housing, whether the family owns the house or not); family context variables (living with parents, parents living together, mother’s age at first pregnancy, openness in the family environment for guidance and conversations about sexuality); behavioral variables (consumption of alcoholic beverages, tobacco, illicit drugs, and if so, age at the beginning of use); variables referring to sources of information (frequency and people/source - parents, family members, teachers, internet or friends); aspects of sexuality (sexual orientation, age at first kiss, relationship situation, initiation of sexual activity, type of sexual intercourse - oral, vaginal and/or anal, and number of partners).

### Data processing and analysis

A database was built with the help of the Excel software, in which the study variables were inserted, according to the answers to the questionnaire. Three spreadsheets were used, one for the first typing, another for the second typing and the third for the coding and decoding dictionary of the variables. To ensure the quality and reliability of the typing process, two typists were trained to perform it, and the data underwent double typing. This procedure allowed comparing the data to verify any inconsistency between the two types, which were corrected when errors were detected.

Data from this study were submitted to descriptive and comparative analysis, using the Statistical Package for the Social Sciences (SPSS - v.17.0 for Windows). For the descriptive statistical analysis of reproductive data, continuous variables were analyzed as measures of central tendency and dispersion, using Student’s t-test for independent samples. Categorical variables were analyzed as absolute and relative frequencies, using the chi-square test; the forms of analysis were also based on the relationship between the participants’ age and the age at the beginning of sexual life, which were described using simple descriptive statistics through the mean, standard deviation and count.

### Ethical aspects

All participants were authorized by their parents or guardians to participate in the research stated in the ICF. From there, the TALE was read together with each participant, as well as the objectives of the study. Participants were guaranteed confidentiality and given the freedom to participate or not, as well as to withdraw at any time, without any harm to their life at school. The study was approved by the Research Ethics Committee (CEP) linked to the National Research Ethics Committee of the National Health Council (CONEP/CNS), with the protocol Certificate of Presentation of Ethical Assessment (CAAE) No. 04953218.5.0000.5393.

## Results

The study had the participation of 85 adolescents, students from the 7th grade of elementary school II to the 3rd year of high school. Of this total, 44 belonged to school 1 and 41 students to school 2. The participants mean age was 14 years ± 2.3. Most participants were female (n=60; 70.6%), self-reported brown and black (n=53; 62.3%) and white (n=28; 32.8%). Regarding the exercise of paid activity, 10 (11.8%) said they worked or had worked at some time in their lives, with a mean age of starting work of 12.3 years ± 3.0). As for housing, 82 participants (96.5%) reported living in urban areas, with the family’s own housing being the most predominant (n=62; 72.9%).

Regarding the family constitution, 45 (52.9%) have their parents living together in the same house, and 38 (44.7%) said that their mothers had their first pregnancy when they were less than 19 years old. Regarding the family environment, 34 (40.0%) reported a closed/very closed environment; 26 (30.6%) neither open nor closed; 19 (22.3%) open/very open to have a dialogue and 6 (7.1%) did not know how to respond.

Regarding behavioral habits, 44 adolescents (50.6%) said they had already started contacting alcoholic beverages, with age at onset ranging between 7 and 17 years, with a mean of 13.5 years ±1.78. Regarding the habit of smoking, 12 (13.8%) reported having had contact with tobacco, and 16 (18.4%) had only had contact with drugs.

Most adolescents (n=69; 81.2%) reported having received information about sexuality at school, 72 (84.7%) received information about STIs at school, and 74 (87.0%) received information about pregnancy.

Regarding sexual orientation, 47 (55.3%) participants reported being heterosexual, 24 (28.2%) bisexual, 2 (2.3%) homosexual, while 12 (14.1%) could not say or did not consider themselves as any of the options. As for relationship status, 18 (21.2%) reported being in a serious relationship, while 14 (16.5%) were dating someone.

Most adolescents 77 (88.5%) reported having kissed on the mouth, with a mean age of 11.60 years ± 2.4, ranging from 6 to 17 years. Table 1 shows the types of sexual practice, considering oral sex and penetration among participants by sex, self-reported color, work and housing type. Among all adolescents participating in the study, 29 (34.1%) reported having had penetrative sex (vaginal, anal or both), another 18 (21.2%) adolescents reported having had oral sex, which constitutes a scenario where 55.3% of adolescents had some type of sexual practice. The practice of vaginal sex was reported by 27 (31.8%) participants, with a mean age of initiation of 14.5 years ± 1.26, ranging from 12 to 17 years. Females were predominant (n=21; 77%), with a self-reported brown color (n=11; 40%). The practice of anal sex was reported by 6 (7.1%) participants, with a mean age of initiation at 14.4 years ± 2.0) ranging from 12 to 17 years, female (n=5; 83.3 %), with self-reported black color (n=3; 50%). Of the 18 (21.2%) adolescents who reported having had oral sex, 17 (94.4%) were female (p=0.0090) and 10 (55%) were self-reportedly brown (Table 1).

**Table 1:** Sociodemographic characteristics related to sexual practices (oral sex and penetrative sex) in two public schools (n=85). Ribeirão Preto, SP, Brazil, 2019-2020

	Oral sex			Penetrative sex		
Variable	n (%)	n (%)	p value	n (%)	n (%)	p value
	No	Yes		No	Yes	
Sex	n=67	n=18		n=56	n=29	
Female	41 (70.7)	17 (29.3)	0.0090*^†^	36 (62.1)	22 (37.9)	0.3270^†^
Male	25 (96.2)	1 (3.8)		19 (73.1)	7 (26.9)	
Mean age	14 (12.15)	15.5 (15.17)	0.0010	13.6 (2.2)	15.3 (2)	0.0010
Self-reported color						
White	24 (85.7)	4 (14.3)	0.2660^‡^	22 (78.6)	6 (21.4)	0.3180^‡^
Black	16 (80)	4 (20)		12 (60)	8 (40)	
Brown	21 (67.7)	10 (32.3)		18 (58.1)	13 (41.9)	
Yellow	05 (100)	-		04 (80)	1 (20)	
Current job						
Yes	7 (70)	3 (30)	0.4350^‡^	4 (40)	6 (60)	0.0830^‡^
No	60 (80)	15 (20)		52 (69.3)	23 (30.7)	
Home						
Own	46 (76.7)	14 (23.3)	0.4510^‡^	42 (70)	18 (30)	0.2150^‡^
Rented	21 (84)	4 (16)		14 (56)	11 (44)	
Street						
Asphalt	58 (77.3)	17 (22.7)	0.6760^‡^	48 (64)	27 (36)	0.4880^‡^
No paving	8 (88.9)	1 (11.1)		7 (77.8)	2 (22.2)	

*p≤0.05; †Pearson’s chi-square test; ‡Fisher’s exact test

The practice of oral sex and penetrative sex was found to be related to a lower search for information in the family; while adolescents who practiced oral sex sought more information from health professionals, this association was not detected in the analysis of penetrative sex (Table 2).

**Table 2:** Sources of information on sex education for adolescents who initiated sexual practices (oral sex and penetrative sex) in two public schools (n=85). Ribeirão Preto, SP, Brazil, 2019-2020

	Oral sex			Penetrative sex		
Variable	n (%)	n (%)	p value	n (%)	n (%)	p value
	No	Yes		No	Yes	
Parents/Guardians	n=67	n=18		n=56	n=29	
Always	14 (82.4)	3 (17.6)	0.3550^†^	13 (76.5)	4 (23.5)	0.4920^†^
Sometimes	27 (71.1)	11 (28.9)		24 (63.2)	14 (36.8)	
Never	23 (85.2)	4 (14.8)		16 (59.3)	11 (40.7)	
Relatives						
Always	9 (81.8)	2 (18.2)	0.0070*^‡^	6 (54.5)	5 (45.5)	0.0500*^‡^
Sometimes	13 (56.5)	10 (43.5)		11 (47.8)	12 (52.2)	
Never	41 (89.1)	5 (10.9)		35 (76.1)	11 (23.9)	
TV						
Always	3 (75)	1 (25)	0.2540^‡^	3 (75)	1 (25)	0.8500^†^
Sometimes	15 (68.2)	7 (31.8)		13 (59.1)	9 (40.9)	
Never	46 (83.6)	9 (16.4)		36 (65.5)	19 (34.5)	
Health Professionals						
Always	4 (44.4)	5 (55.6)	0.0100*^‡^	4 (44.4)	5 (55.6)	0.0620^‡^
Sometimes	24 (75)	8 (25)		17 (53.1)	15 (46.6)	
Never	34 (89.5)	4 (10.5)		29 (76.3)	9 (23.7)	
Friends						
Always	16 (66.7)	8 (33.3)	0.0620	12 (50)	12 (50)	0.0960
Sometimes	26 (76.5)	8 (23.5)		22 (64.7)	12 (35.3)	
Never	20 (95.2)	1 (4.8)		17 (81)	4 (19)	
Internet						
Always	12 (66.7)	6 (33.3)	0.2140	11 (61.1)	7 (38.9)	0.8610
Sometimes	25 (78.1)	7 (21.9)		21 (65.6)	11 (34.4)	
Never	28 (87.5)	4 (12.5)		22 (68.8)	10 (31.2)	
teachers						
Always	8 (80)	2 (20)	0.4090	6 (60)	4 (40)	0.2520
Sometimes	22 (71)	9 (29)		17 (54.8)	14 (45.2)	
Never	32 (84.2)	6 (15.8)		28 (73.7)	10 (26.3)	

*p≤0.05; †Pearson’s chi-square test; ‡Fisher’s exact test

An association was found between adolescents’ sexual practices (oral and penetrative sex) and alcohol, tobacco and drug use (p<0.05), as shown in Table 3.

**Table 3:** Adolescents’ risk habits related to sexual practices (oral sex and penetrative sex) in two public schools (n=85). Ribeirão Preto, SP, Brazil, 2019-2020

	Oral sex			Penetrative sex		
Variable	n (%)	n (%)	p value	n (%)	n (%)	p value
	No	Yes		No	Yes	
Alcohol use	n=67	n=18		n=56	n=29	
No	41 (95.3)	2 (4.7)	0.0010*^†^	35 (81.4)	8 (18.6)	0.0020*^†^
Yes	26 (61.9)	16 (38.1)		21 (50)	21 (50)	
Cigarette use						
No	52 (83.9)	10 (16.1)	0.0040*^‡^	45 (72.6)	17 (27.4)	0.0010*^‡^
Yes	5 (41.7)	7 (58.3)		2 (16.7)	10 (83.3)	
Drug Use						
No	58 (84.1)	11 (15.9)	0.0360*^‡^	52 (75.4)	17 (24.6)	0.0010*^‡^
Yes	9 (56.2)	7 (43.8)		4 (25)	12 (75)	
Drug type						
Does not use	57 (83.8)	11 (16.2)	0.0160*^‡^	52 (76.5)	16 (235)	0.0010*^‡^
One	2 (33.3)	4 (66.7)		1 (16.7)	5 (83.3)	
More than one	8 (72.7)	3 (27.3)		3 (27.3)	8 (72.7)	

**p≤0.05; †Pearson’s chi-square test; ‡Fisher’s exact test

## Discussion

The data from this study made it possible to characterize the initiation of sexual practices in adolescence, such as oral, vaginal and anal sex, in addition to evaluating the adolescents’ family, socioeconomic context and risk habits, including the use of substances such as alcohol, tobacco and drugs and their association with sexual initiation.

Regarding sexual orientation, approximately one third of the adolescents reported being bisexual and/or homosexual. Adolescents belonging to sexual minorities are more vulnerable and more exposed to risk factors than heterosexual adolescents^12^. Studies with homosexual and bisexual adolescents have detected a higher risk of acquiring STIs, sexual practices after drinking alcohol, unprotected sex, violence, sexual abuse, teenage pregnancy, early sexual initiation (<13 years), greater number of sexual partners, use of illicit substances, symptoms of depression and other mental health problems, in addition to lack of family support and school bullying^12^ ^)-(^ ^14^. The needs and specificities of this population group deserve a focused look from public policies and health practices.

The most frequent sexual behaviors and practices observed in the present study were kissing, followed by vaginal, oral and anal sex and a mean age of 14.5 years for penetrative sex. Brazilian studies report that 25-47.3% of adolescents have already had sexual intercourse^5^ ^),(^ ^7^ ^),(^ ^9^ ^)-(^ ^10^ ^),(^ ^15^, with a mean of initiation at 14 years^7^ ^),(^ ^9^. This study identified an association between the practice of oral sex and being a female teenager, which is different from another Brazilian study that detected a higher prevalence of this practice in adolescents and young males, and the more frequent practice of exclusive vaginal penetration among women^14^. A Dutch longitudinal study detected three trajectories of sexual development in adolescence: i) non-active sexual trajectory, with adolescents with less involvement in sexual activity; ii) gradually active (linear) sexual trajectory, with a sexual progression of practices that evolve from minor to major sexual contact; iii) and a fast (non-linear) active sexual trajectory, with adolescents who show an evolution of sexual practices in a short period of time^16^. The latter is related to adolescents’ social factors and is predominantly characterized by the inconsistent use of contraceptive methods, possibly due to the shorter time for reflection on the acts^16^.

A study carried out in the United States showed that, of the women who started vaginal sexual practice first, 31.4% reported teenage pregnancy. Among women who had vaginal and oral sex concomitantly, 20.5% reported adolescent pregnancy, while among those who only initiated oral-genital sexual intercourse first, only 7.9% reported adolescent pregnancy^17^. Possibly, initiating the practice of oral-genital sex and with an interval of one year to change to other behaviors may allow adolescents to develop better skills related to sexual planning and the ability to negotiate decisions regarding contraception with partners as they progress to vaginal sex intercourse^16^ ^)-(^ ^17^. This is possibly in line with the data from the present series, which detected that adolescents who practiced oral sex sought more information related to sex education with health professionals.

The family is the ideal context for the formation of individuals, as it is the main means of acquiring the values necessary to live in society. Although parents have greater proximity and regular contact with their children^18^, there may be reluctance to address sexuality and create communication barriers^19^, which may influence adolescents to seek information from other family members, and even among peers. Many parents may feel unprepared and uncomfortable in approaching the topic, due to the influence of cultural norms and religion^18^ ^)-(^ ^19^. The approach to sexuality often takes place with a focus on biological aspects related to contraception and STIs and human immunodeficiency virus (HIV) infection prevention, as well as the educational and economic repercussions and on the adolescent’s reputation in the community^19^, with an imposing and punitive characteristic.

The family’s lack of open dialogue about sexuality within spaces where teenagers feel safe and welcomed suggests that they should seek information that prepares them for the beginning of sexual life early and in unfavorable places. The literature points out that in contemporary western urban society, adolescents have extensive contact with references to the great value of sex and sexual freedom, through the media and the internet^20^. Learning about the body often happens alone, with the search for information on social networks, magazines aimed at teenagers and, occasionally, in books^21^, which was not a finding of this study. However, it is known that insufficient dialogue about sexual health between parents and adolescents is one of the factors that contribute to high rates of teenage pregnancy and STIs, including HIV infection^19^. It is worth mentioning that only the provision of information is incipient to promote preventive behaviors, it becomes relevant to continuously promote adolescents’ reflection, critical thinking and awareness about self-care.

There was an association of adolescents’ sexual practices (oral and penetrative sex) with alcohol, tobacco and drug use. A US study showed that non-consumption of alcohol among male and female adolescents before the last sexual intercourse was associated with greater use of condoms^22^. On the other hand, a Brazilian survey showed an association with unprotected sexual practices by adolescents who use alcohol, drugs and tobacco^5^. It is noted that the consumption of such substances is related to early sexual initiation^10^ ^),(^ ^14^ and multiple sexual partners^14^ ^),(^ ^23^ and increases the probability of unprotected sexual practices^5^ ^),(^ ^14^ ^),(^ ^22^ ^),(^ ^24^, with a higher risk of acquiring STIs and HIV^10^ ^),(^ ^22^ ^),(^ ^24^ ^)-(^ ^26^, teenage pregnancy^23^ ^)^ and sexual violence^25^ ^)-(^ ^26^. There is a greater consumption of drugs in male adolescents/young people, with marijuana being the main use, as well as a greater tendency to engage in sexual practices after drug consumption^14^.

In Brazil, Law No. 13,106 of March 17, 2015^27^ criminalizes the sale of alcoholic beverages to children and adolescents, but alcohol consumption is the psychoactive substance most frequently used by them^24^. Substance use causes deleterious effects on adolescents’ physical, mental and social health, and can even lead to death, in addition to significant repercussions in adulthood^24^. With regard to sexual and reproductive health, alcohol and/or marijuana consumption in adolescence is associated with greater sexual behaviors with risk for unfavorable sexual outcomes in adulthood, such as acquiring STIs in young adults, establishing multiple sexual partnerships, unprotected sex and unintended pregnancies^23^. In homosexual and bisexual populations, there is a high early consumption of alcohol and tobacco in adolescence, associated with abuse and dependence on these substances in adulthood^28^. Furthermore, the consumption of alcohol, tobacco and drugs in adolescents is associated with school problems, including absenteeism, school dropout, difficulties in teaching and learning, and school violence^24^ ^),(^ ^29^.

With this problem, the central role of Primary Health Care (PHC) and nurses in strengthening public policies and in the use of care technologies in comprehensive and humanized healthcare for adolescents and their families is highlighted, based on the recognition of adolescents as subjects of human, sexual and reproductive rights. On the collective level, health promotion and disease prevention strategies are highlighted, by articulating education and health practices to achieve sensitivity, awareness and mobilization, in order to encourage subjects to relate, express themselves and generate conscious behaviors^30^ ^)^ in making choices that are more favorable to health and developing autonomy and self-care regarding the prevention of unplanned pregnancy, STIs and AIDS; right to sexual and reproductive health; preventing alcohol, tobacco and other drug use; prevention of violence, promotion of a culture of peace and human rights^1^ ^),(^ ^31^.

In this context, the importance of extramural actions emerges, with the integration of social facilities, such as schools, associations, among others, in addition to the involvement of parents, families and the community. Along these lines, the Health at School Program (PSE) stands out, established by Decree No. 6,286, of December 5, 2007, which aims at the permanent integration and articulation of education and health, with the objective of offering a range of actions for the prevention, promotion and health care of children, adolescents and young people in public basic education in the Brazil^32^. The holding of debates, conversation circles, workshops or culture circles represent teaching strategies and pedagogical methodologies that can be used by nurses in actions in schools. Such strategies allow the active participation of adolescents, in addition to promoting their visibility, expression, autonomy, contributing to their critical and reflective training and to their empowerment^30^.

On the individual level, the role of the nursing consultation (NC) stands out as a technological care strategy and activity incorporated into the actions of nurses in primary care^33^ ^)-(^ ^34^, based on systematized actions that make up the nursing process, and which provide autonomy and professional empowerment, in addition to being a space for care with potential to expand access and resolution in PHC^34^. NC represents a privileged space for promoting adolescent and family health, in addition to providing the identification of adolescents’ main complaints and demands, usually related to sexual health, issues inherent to mental health such as the presence of depressive symptoms and substance use^35^.

At this moment, welcoming, sensitive listening^1^, confidentiality^35^ ^)^ and the creation of bonds^36^ ^)^ provide an opportunity for dialogue and clarification of doubts^1^ ^),(^ ^36^, to promote reflections on the importance of affection and pleasure in love relationships and to warn about vulnerabilities and risk situations for violence and/or sexual exploitation^1^, among other demands according to the adolescent’s health needs, specificities, singularities and experiences. Thus, NC allows adolescents to take a leading role in their choices^36^, as well as counseling on responsible and safe sexual and health practices. It is recommended to advise adolescents about internal and external condom use as an indispensable practice in the prevention of STIs and HIV infection^1^ ^)^ in all consultations.

Also, the role of school nurses in creating and enhancing spaces for health promotion at school is highlighted, with the appreciation of sex education, participation in the planning and development of curricular activities in conjunction with teachers and coordinators, in addition to communication with students, parents, families and the community^37^, counseling on issues related to sexual orientation and care for adolescents belonging to sexual and gender minorities^38^, identification of children and adolescents at risk or victims of commercial sexual exploitation^39^, as well as the implementation of measures to prevent alcohol, tobacco and drug consumption, and the identification and referral of adolescents who use substances for treatment^40^.

The study has limitations because it deals with a convenience sample. However, the results contribute to nursing care practices, highlighting the importance of risky habits such as the use of alcohol, tobacco and drugs and their association with sexual initiation. The study also highlighted the lack of dialogue with family members about the subject and the diversity of sexual practices performed by adolescents, and the relevance of a careful look and approach to gender and sexual orientation issues and their possible implications for the health of this population. The aspects listed in this study demonstrate sensitive points for designing interventions for health promotion and prevention of diseases and injuries for adolescents.

## Conclusion

The results of this study allowed us to describe the diversity of sexual practices among adolescents, with a predominance of vaginal sex, followed by oral and anal sex, with a tendency towards early initiation, gender influence and the association of alcohol, drug and cigarette use with sexual activities. These findings emphasize the role of nurses in planning and carrying out health education interventions within the PHC scope, in integration with schools, families and the community, regarding the promotion, protection and comprehensive care of adolescent health. Another connection was in the initiation of safer sex with the family context, elucidating the fact that a more welcoming family environment, open to dialogue, generates an important impact on the decision process of having sexual relations in more or less safe ways and needs to gain space in public policy-making aimed at adolescent health.
